# Case Report: Unusual Presentation of Myositis Ossificans of the Elbow in a Child Who Underwent Excessive Postoperative Rehabilitation Exercise

**DOI:** 10.3389/fped.2021.757147

**Published:** 2021-11-12

**Authors:** Jin Cao, Hua Jiang Zheng, Jing Hua Sun, Huan Ye Zhu, Chao Gao

**Affiliations:** ^1^Department of Orthopedics, Ningbo Sixth Hospital, Ningbo, China; ^2^Department of Pathology, Ningbo Diagnostic Pathology Center, Ningbo, China

**Keywords:** myositis ossificans, excessive postoperative rehabilitation exercise, child, conservative treatment, bone health

## Abstract

Traumatic myositis ossificans (MO) is an unusual complication after muscle injury and is predominantly seen in young adults and adolescents. Pediatric MO cases are even rarer. We report an 8-year-old girl who was diagnosed with a lateral humeral condyle fracture. She was treated surgically, and her elbow joint was fixed with plaster. Rehabilitation exercise was administered 1 month after the operation. Due to the wrong exercise method, a palpable bony mass appeared around the elbow 1 month later. The clinical radiological diagnosis showed MO, and conservative treatment was administered. After 3 years of follow-up, the affected limb functioned well, with no sign of recurrence. Here, we report this long-term follow-up case of MO resulting from excessive rehabilitation exercise.

## Introduction

Myositis ossificans (MO) is a benign tumor-like lesion, which is predominantly seen in young adults and adolescents. Pediatric MO cases are even rarer (1). Approximately 75% of the cases are caused by trauma and are more common in adults than in children (2). It occurs in areas under constant high risk of injuries, such as the brachialis, quadriceps femoris, and thigh adductor muscles (3). Patients typically present with pain and restricted range of motion following trauma or overuse (4). The probable etiological causes include repetitive minor mechanical injuries, ischemia, and inflammation (5). The pathological and imaging manifestations of MO may change with its developmental stage. The early-stage (<4 weeks) MO mainly consists of fibroblasts and myofibroblasts. In the middle stage (4–8 weeks), MO is characterized by the presence of osteoblasts and gradually becomes a mature bone tissue. In the mature period (>8 weeks), the mass includes mature lamellar bone (6, 7). Computed tomography (CT) scan gives better results in the early stage than in later stages (5). Additionally, although results from magnetic resonance imaging (MRI) may be confusing in the early stage, a “striate pattern” or “checkerboard-like pattern” increases the possibility of MO (8). After 4 weeks, as the peripheral calcifications gradually appear, X-ray radiography can be effective in the diagnosis (6). A biopsy can accurately diagnose MO, however such invasive manipulation may cause further bone hyperplasia and worsen the prognosis (9). This article reports a pediatric patient with MO caused by excessive postoperative rehabilitation exercise. Conservative treatment with long-term follow-up was effective.

## Case Report

**Figure 1:**
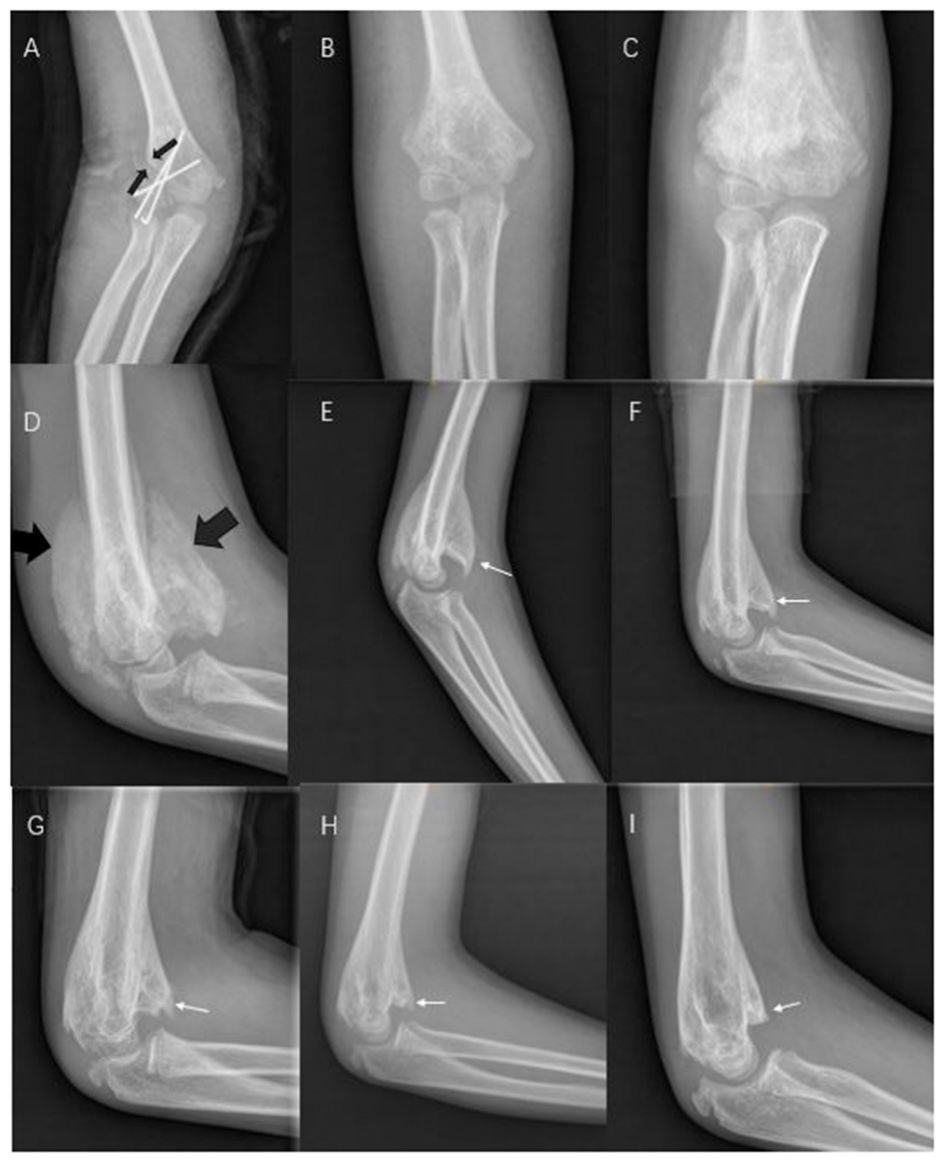
**(A)** Postoperative day 1 (black arrows show the fracture). **(B)** Plaster and Kirschner wire were removed after 1 month. **(C,D)** After 1 month of functional exercise (black arrows show the bone hyperplasia of the myositis ossificans [MO]). **(E–I)** The MO gradually disappeared, and merged with the humerus (white arrows show that the hyperplastic bone affecting elbow flexion gradually disappears).

An 8-year-old girl complained of pain, swelling, and aggravated restricted movement in the right elbow. She was injured in a fall while riding her bicycle an hour prior. Radial and ulnar artery pulses were palpable, the ulnar joint of the humerus was stable, and the medial condyle and supracondyle were painless. However, she had pronounced tenderness in the lateral condyle. X-ray radiography revealed a fracture in the lateral humeral condyle. The elbow was immediately immobilized with a cast. After 2 days, an experienced pediatric orthopedist treated the fracture with open reduction and performed internal fixation under brachial plexus anesthesia and with the patient placed in the supine position while the orthopedist used a pneumatic tourniquet. Briefly, a longitudinal skin incision (~4 cm) was made directly on the lateral condyle of the humerus on the outside of the elbow joint under aseptic conditions. After separating the skin and fascia layer by layer, the fracture ends were exposed. After clearing the congestion and reducing the incarcerated soft tissue, three K-wires were used to fix the fracture. The results from intraoperative fluoroscopy showed a good reduction. The lateral collateral ligament was examined to confirm its integrity immediately. Postoperatively, a long-arm posterior splint was placed with the elbow flexed 90 degrees (Figure 1A). The operation went smoothly, and the patient was discharged after 1 week. Her right arm was splinted for 4 weeks after the surgery. After her discharge from the hospital, she was reviewed at the outpatient clinic. The plaster was removed in the outpatient operating room 4 weeks later, and the Kirschner wire was removed (Figure 1B). The elbow joint movement was limited with the flexion and extension of approximately 50° (range 70–110°). The Broberg-Morrey score was 44, and thus functional exercise was administered. However, the patient presented to the outpatient clinic with pain in the elbow joint after 1 month because of excessive exercise. Her mother had noticed a palpable mass inside the elbow, which was found to be a bone-like structure *via* X-ray radiography (Figures 1C,D). Since the patient displayed no obvious neurological symptoms, conservative treatment with long-term follow-up was implemented. We also corrected the maladaptive recovery. Specifically, to prevent further bone hyperplasia caused by exercise, the elbow joint was fixed with a cast for 2 weeks. When the patient complained of pain in the elbow joint, analgesics (4% ibuprofen suspension drops) were administered. After the plaster was removed, the patient could perform slow active exercises for the elbow joint function without any pain. The patient was followed up five times in total, and the follow-up time points were 2018/10 (Figure 1E), 2018/12 (Figure 1F), 2019/3 (Figure 1G), and 2019/7 (Figure 1H). The results from X-ray radiography during the postoperative follow-up examinations revealed that the MO gradually disappeared and merged with the humerus. The last follow-up was 3 years after the surgery (Figure 1I), and clinical examination revealed no evidence of recurrence. The patient displayed elbow joint movement with the flexion and extension of approximately 120° (range 0–120°). The Broberg-Morrey score was 97.

## Discussion

MO is predominantly seen in young adults and adolescents and is most commonly secondary to trauma. Although the exact etiology remains unclear, patients typically present with pain and restricted range of motion following trauma or overuse (10). In this article, we show that excessively exercising the elbow joint after surgery can cause MO development in the joint. Lesions are known to usually occur in the large muscle groups of the thighs and upper limbs. The brachialis, quadriceps, femur, and adductors of the thigh are the most commonly affected areas. Physical examination revealed that this patient had a hard tender mass in the soft tissue, but the density of calcification was visible on the X-ray image after several weeks.

Due to the bone hyperplasia early during the disease, such masses may be mistaken for osteosarcoma or soft-tissue tumors (6). Through X-ray radiography, we observed an intact cortex and dense calcification around the lesion. Osteosarcoma is often accompanied by periosteum hyperplasia, bone-cortex destruction, and unclear borders. After approximately 10 weeks of growth, the mass spontaneously stagnated and then subsided, as evidenced *via* radiographic monitoring. Thus, the mass was not a tumor.

Osteochondral fractures (TRASH lesions) often require surgical intervention (11). As the articular part of the lateral condyle is ossified during adolescence, the cartilage fracture cannot be detected *via* X-ray radiography, whether it is accompanied by any TRASH lesion should be determined. MRI or arthrography can be more informative than X-ray radiography in the assessment for a cartilage fracture. In clinical practice, the gap of a lateral condyle fracture is often used to determine whether surgical treatment is required. The fracture is stable when the fracture gap is <2 mm, often without osteochondral fractures, and plaster fixation is often used as the treatment modality. However, the lateral condyle of this patient was significantly displaced (>2 mm), and thus the fracture gap was deemed unstable and needed closed reduction along with K-wire fixation (12). During the operation, an osteochondral fracture of the articular surface was also detected.

An intense fibrocartilagenous callus can also develop in lateral condyle fractures, albeit rarely. Such cases are often accompanied by nonunion and lack adequate immobilization or stability to progress to the union. They are common in the conservative treatment of pediatric clavicle fractures (13). Studies have shown that brain injury with limb fracture, can also be accompanied by limb fracture intense callus formation (14). This maladaptive formation also manifests as a palpable mass near a fracture, and imaging analyses show that the hyperporosis and connected to the fracture (15). Imaging analyses show no obvious bone density in early-stage MO, and bone formation gradually appears in the middle and late stages of the disorder (8). Since MO is mostly located in muscles, it has no obvious association with fractures (Figure 1D).

MO is typically benign and self-limiting (16). Thus, surgical removal of calcified lesions is not necessary for children unless the lesions are painful or hinder movement. Desai (17) treated a child with a popliteal space lesion *via* conservative treatment. Over a period of 14 months, radiographs showed increasing ossification of the mass, which required operative treatment. Kanthimathi et al. (18) reported a 13-year-old boy with MO in his left elbow, which was immobilized for 4 weeks because his left upper limb suffered significant trauma. The stiffness of the elbow lasted for 14 months. Eventually, excision was performed. Li (19) reported a 9-year-old elbow MO resulting from cellulitis. Due to the pain, swelling, and aggravated restricted movement, the mass was eventually excised. In the report of Sferopoulos (20), 22 patients with MO, four of whom had undergone poor conservative treatment, including one case on the chest wall, one case on the pelvis, and two cases on the thigh, were treated with surgery. The remaining 18 patients were treated conservatively and recovered well. Here, we reported a case of excessive postoperative rehabilitation exercise complicated with MO. Since the patient had no neurological symptoms, we adopted conservative treatment and followed up for a long time. The patient recovered well after surgery, and the MO gradually disappeared.

In conclusion, this article reports a rare case of pediatric MO in the elbow, which we successfully treated *via* conservative treatment. Consistent with the aforementioned reports, our observations indicate that conservative management can be an effective first-line strategy.

## Limitation

The most important limitation of this study is that the follow-up time may be too short to predict the long-term prognosis. Since the recovery from MO takes a long time, the 3-year follow-up duration may not fully reflect the course of the disease. In addition, since this study involved only one patient, the conclusions may not be universal. The lack of an early CT examination of the fracture is another limitation of this study, CT examination can obtain a more accurate diagnosis with less risk.

## Informed Consent

The parents of the patient in this study were informed about the management of MO. They chose conservative treatment and signed a consent form for the publication of the study results involving their child and for the long-term follow-up.

## Data Availability Statement

The original contributions presented in the study are included in the article/supplementary material, further inquiries can be directed to the corresponding author/s.

## Ethics Statement

Written informed consent was obtained from the minor(s)' legal guardian/next of kin for the publication of any potentially identifiable images or data included in this article.

## Author Contributions

HJZ, JHS, and HYZ collected the data. JC wrote the first draft of the manuscript. CG contributed to data interpretation, edited the manuscript, and approved the final version. All authors were involved in the conception of the study.

## Conflict of Interest

The authors declare that the research was conducted in the absence of any commercial or financial relationships that could be construed as a potential conflict of interest.

## Publisher's Note

All claims expressed in this article are solely those of the authors and do not necessarily represent those of their affiliated organizations, or those of the publisher, the editors and the reviewers. Any product that may be evaluated in this article, or claim that may be made by its manufacturer, is not guaranteed or endorsed by the publisher.
